# Learning from each other in the COVID-19 pandemic

**DOI:** 10.12688/wellcomeopenres.15973.2

**Published:** 2021-10-12

**Authors:** Anna C. Seale, Maryirene Ibeto, Josie Gallo, Olivier le Polain de Waroux, Judith R. Glynn, Jenny Fogarty

**Affiliations:** 1 UK Public Health Rapid Support Team, London School of Hygiene & Tropical Medicine and Public Health England, London, UK; 2 Faculty of Epidemiology and Population Health, London School of Hygiene & Tropical Medicine, London, WC1E 7HT, UK; 3 Centre for Excellence in Learning and Teaching, London School of Hygiene & Tropical Medicine, London, WC1E 7HT, UK

**Keywords:** COVID-19, education, infodemic, MOOC

## Abstract

The increase in cases of coronavirus disease 2019 (COVID-19) worldwide has been paralleled by increasing information, and misinformation. Accurate public health messaging is essential to counter this, but education may also have a role. Early in the outbreak, The London School of Hygiene & Tropical Medicine partnered with FutureLearn to develop a massive open online course (MOOC) on COVID-19. Our approach was grounded in social constructivism, supporting participation, sharing uncertainties, and encouraging discussion. The first run of the course included over 200,000 participants from 184 countries, with over 88,000 comments at the end of the three-week run. Many participants supported each other’s learning in their responses and further questions. Our experience suggests that open education can complement traditional messaging, potentially providing a sustainable approach to countering the spread of misinformation in public health.

Worldwide connectivity has facilitated spread of the virus causing COVID-19, and this has been almost paralleled by the spread of information and misinformation ^1^. Sharing accurate information, for example through reliable, trusted, social media accounts and websites, as well as responding to misinformation with MythBusters is important ^2^. However, education may also play an in important role, to support health literacy, and early on in the outbreak of COVID-19, The London School of Hygiene & Tropical Medicine (LSHTM) agreed to develop a massive open online course (MOOC) on COVID-19, in partnership with FutureLearn, as described here, and following courses on previous outbreaks such as Ebola and Zika.

MOOCs offer an opportunity for wide participation, but there is a challenge to engage at scale, and maintain participation, and MOOCs can have high drop-out rates (between 95–98%) ^3^. With these challenges in mind, we brought together a multi-disciplinary group to share and discuss, at pace, what a MOOC on COVID-19 could look like, in terms of approach, content and style. We grounded our MOOC in educational theory and utilised social constructivist principles ^4^. The pedagogical approach is considered to be the most important dimension in the quality of a MOOC ^4^, and constructivist approaches, placing the learner at the centre, have explicit expectations for engagement, to better support deep learning, and the ability to appraise information critically ^5 ^; important aspects for public health literacy.

We began with consideration of who the intended learners might be, and what they would know, and we structured the course simply, in terms of what was known at that time about COVID-19, what a public health response may look like in various settings, and what research was needed to understand more. Within this, each step of the course had defined intended learning outcomes, which contributors were asked to address. We included international contributions to share global perspectives on the pandemic. To support engagement and maximise opportunities provided by the platform, we used varied formats including short video lectures, audio interviews, articles and quizzes. To maximise accessibility for learners we included subtitles and transcripts, in several languages. A key part of the engagement occurred after each part of the course, where we encouraged participation and engagement through specific questions posed by course facilitators.

At the outset we didn’t know what the uptake to the course, in the context of a pandemic, would be, or who would enrol. There was also the concern that evidence would have moved on before the course had even started, as materials were developed 3–4 weeks before the course start (to allow time for translation), and the number of publications and preprints on COVID-19 was increasing rapidly. In a time of heightened public anxiety, by taking a participatory approach to the course, which included sharing uncertainties in terms of what was known, and encouraging discussion and questioning, we, and our institution, took a substantial risk. It could, for example, have resulted in the mass sharing of extreme views by participants. In subsequent iterations it still could. However, as teachers and researchers we work to both learn more, and to share this learning. We are supported institutionally with the academic freedom to do so; there was no corporate “sign-off” from LSHTM.

For the first run of the course, starting in late March, we had very high uptake, with over 170,000 participants in the first three weeks, and over 200,000 in total in the first run, from 184 countries. We also noted very high levels of engagement, with over 88,000 comments, and many people supporting each other’s learning in their responses and further questions, demonstrating peer-learning and connectivism ^3^. Within LSHTM we set up a system to respond to as many queries as possible, even with a small team, to provide feedback and guidance to learners. Non-technical hosts read through discussion fora and collated queries and synthesised areas of interest for each course step, which academics then responded to, helping to keep the course current.

Much of the feedback on the course, captured through course comments and the end of course survey, has been positive, highlighting the opportunity to gain new knowledge and skills, and in the first run, just over a third of those enrolled completed the course. Running the course, we felt that developing participants’ own learning, and providing a space where ideas – and fears – could be expressed and addressed was central to their learning and participation. But what surprised us was the level of expressed gratitude, perhaps reflecting a gap in such educational opportunities in public health.

Our experience suggests that MOOCs can be developed at pace, with an appropriately skilled and motivated team, to support learning in a public health emergency, and maintain quality across pedagogical, organisational, technological, and social domains ^6^. From a public health perspective, we consider that building individuals’ own capacity to question, to query the data, reports and guidance is essential, and complementary to the circulation of accurate public health information. Further investigation of the role of MOOCs to support health literacy, which has an established role in the context of patient behaviour change ^7^, should be considered in the particular context of infectious disease outbreaks ^2^, to better understand and inform public health practice.

## Data availability

No data are associated with this article.
