# Supplementary material for: Learning from each other in the COVID-19 pandemic
Source: Wellcome Open Res. 2021 Oct 12;5:105. Originally published 2020 May 26. [Version 2] doi: 10.12688/wellcomeopenres.15973.2 (PMC8602955; doi:10.12688/wellcomeopenres.15973.2)
Supplement: Peer review history

# Peer review: Reviewer response for version 2

**DOI:** 10.21956/wellcomeopenres.19089.r46370

**Published:** 2021-11-18

**Authors:** Ana Pérez-Escoda

**Affiliations:** 1 Department of Communication, University Antonio de Nebrija, Madrid, Spain

The authors have improved their proposal in response to the suggestions made, and it is therefore considered that the proposal meets the requirements for publication and dissemination. Congrats for the authors!

Does the article adequately reference differing views and opinions?

No

Are all factual statements correct, and are statements and arguments made adequately supported by citations?

Partly

Is the Open Letter written in accessible language?

Yes

Where applicable, are recommendations and next steps explained clearly for others to follow?

Not applicable

Is the rationale for the Open Letter provided in sufficient detail?

Partly

Reviewer Expertise:

New learning approaches, digital literacy, digital skills, digital communication, learning methodology.

I confirm that I have read this submission and believe that I have an appropriate level of expertise to confirm that it is of an acceptable scientific standard.

# Peer review: Reviewer response for version 1

**DOI:** 10.21956/wellcomeopenres.17518.r41135

**Published:** 2021-06-01

**Authors:** Cristina Pulido Rodriguez

**Affiliations:** 1 Department of Journalism and Communication Sciences, Autonomous University Of Barcelona, Bellaterra, Spain

The open letter "Learning from each other in the COVID-19 pandemic" accomplishes this type of submission instructions, but there are improvements that could increase the impact of this contribution. The open letter describes the MOOC on Covid-19 developed and how many people were engaged and what they learnt in general terms. But, the following specific instruction is not well addressed "The rationale for the Open Letter should be explained, including an outline of existing challenges and the purpose of the letter, different views and opinions in the field should be referenced appropriately". The open letter describes more the specific experience obtained with the "MOOC on Covid-19", but a general overview on this topic with different views is missed. Another improvement to be considered is to illustrate results with specific examples, not as a research paper because the open letter does not require this, but it could help to identify the real benefits and improvements of applying this type of MOOC.

Does the article adequately reference differing views and opinions?

Partly

Are all factual statements correct, and are statements and arguments made adequately supported by citations?

Partly

Is the Open Letter written in accessible language?

Yes

Where applicable, are recommendations and next steps explained clearly for others to follow?

Partly

Is the rationale for the Open Letter provided in sufficient detail?

Partly

Reviewer Expertise:

My areas of research are: communication sciences, education and sociology.

I confirm that I have read this submission and believe that I have an appropriate level of expertise to confirm that it is of an acceptable scientific standard, however I have significant reservations, as outlined above.

# Peer review: Reviewer response for version 1

**DOI:** 10.21956/wellcomeopenres.17518.r42469

**Published:** 2021-03-01

**Authors:** Ana Pérez-Escoda

**Affiliations:** 1 Department of Communication, University Antonio de Nebrija, Madrid, Spain

The Open letter states that education would be one of the main benchmarks for avoiding misinformation by presenting the impact of the MOOC developed by the London School of Hygiene & Tropical Medicine partnered with FutureLearn. Although the topic is certainly relevant and timely it is suggested to add more references to support the arguments that could build up a more suitable and relevant theoretical framework.

 It is suggested to add some literature about MOOC focusing on specific features, about learning communities, which is a specific research topic in the open letter; and, also it is recommend some literature about misinformation, authors state that education is in the basis of facing misinformation but it is barely explained. This is essential for a better understanding of the main statements contained in the open letter.

 The authors provided the rationale for the Open Letter however they should also outline existing challenges in the field for instance the level of dropout in MOOCs. The Open Letter just focus on descriptive numbers in terms of participants, countries or comments, but how many of that first run participants finished the MOOC?

 Facing misinformation is challenging in different dimensions and globally speaking as acknowledged by WHO with the “infodemic” problem. In this regard, it should be better if authors are less enthusiastic with their statements such as: ”By using open platforms to build communities of learners, to share the values of academia through education, we can support sustainability in countering the spread of misinformation”. A more cautious and realistic wording is suggested, underlining the value of these initiatives in alleviating part of the problem.

Does the article adequately reference differing views and opinions?

No

Are all factual statements correct, and are statements and arguments made adequately supported by citations?

Partly

Is the Open Letter written in accessible language?

Yes

Where applicable, are recommendations and next steps explained clearly for others to follow?

Not applicable

Is the rationale for the Open Letter provided in sufficient detail?

Partly

Reviewer Expertise:

New learning approaches, digital literacy, digital skills, digital communication, learning methodology.

I confirm that I have read this submission and believe that I have an appropriate level of expertise to confirm that it is of an acceptable scientific standard, however I have significant reservations, as outlined above.

# Peer review: Reviewer response for version 1

**DOI:** 10.21956/wellcomeopenres.17518.r42470

**Published:** 2021-02-22

**Authors:** Silvia Lizett Olivares Olivares

**Affiliations:** 1 Tecnologico de Monterrey, School of Medicine and Health Sciences, Monterrey, Mexico

The open letter describes a MOOC with high impact on participants learning about an important topic. The purpose to educate the public is important to address their concerns and misinformation about COVID-19.

 I would recommend supporting information with MOOC literature, specially about quality dimensions, design and specific features. 

 Results from the MOOC are only oriented by numbers (participants, countries, or comments). I recommend describing the type of feedback received from quotes, or satisfaction measures.

Does the article adequately reference differing views and opinions?

Partly

Are all factual statements correct, and are statements and arguments made adequately supported by citations?

Partly

Is the Open Letter written in accessible language?

Yes

Where applicable, are recommendations and next steps explained clearly for others to follow?

Yes

Is the rationale for the Open Letter provided in sufficient detail?

Yes

Reviewer Expertise:

Higher education research, especially health sciences education.

I confirm that I have read this submission and believe that I have an appropriate level of expertise to confirm that it is of an acceptable scientific standard, however I have significant reservations, as outlined above.
